# GAPDH Pseudogenes and the Quantification of Feline Genomic DNA Equivalents

**DOI:** 10.1155/2013/587680

**Published:** 2013-04-28

**Authors:** A. Katrin Helfer-Hungerbuehler, Stefan Widmer, Regina Hofmann-Lehmann

**Affiliations:** Clinical Laboratory, Vetsuisse Faculty, University of Zurich, Winterthurerstrasse 260, 8057 Zurich, Switzerland

## Abstract

Quantitative real-time PCR (qPCR) is broadly used to detect and quantify nucleic acid targets. In order to determine cell copy number and genome equivalents, a suitable reference gene that is present in a defined number in the genome is needed, preferably as a single copy gene. For most organisms, a variable number of glyceraldehyde-3-phosphate dehydrogenase (GAPDH) pseudogenes have been reported. However, it has been suggested that a single-copy of the GAPDH pseudogene is present in the feline genome and that a GAPDH assay can therefore be used to quantify feline genomic DNA (gDNA). The aim of this study was to determine whether one or more GAPDH pseudogenes are present in the feline genome and to provide a suitable alternative qPCR system for the quantification of feline cell copy number and genome equivalents. Bioinformatics and sequencing results revealed that not just one but several closely related GAPDH-like sequences were present in the cat genome. We thus identified, developed, optimized, and validated an alternative reference gene assay using feline albumin (fALB). Our data emphasize the need for an alternative reference gene, apart from the GAPDH pseudogene, for the normalization of gDNA levels. We recommend using the fALB qPCR assay for future studies.

## 1. Introduction

Fluorescence-based quantitative real-time PCR (qPCR) is a highly sensitive method for the detection and quantification of nucleic acids. Due to its conceptual simplicity, sensitivity, specificity, and speed, qPCR applications can be found in a variety of fields, including medicine and the life sciences [1, 2]. In clinical diagnostics, qPCR is broadly used for the detection and quantification of bacterial and viral loads, gene dosage determination, cancer diagnostics, and applications in forensic medicine [3–7].

To assess the cell number present in a PCR reaction, the coanalysis of suitable reference genes is crucial. Such reference genes should be single-copy number genes and should not frequently undergo genetic alterations, to allow the accurate normalization of genomic DNA (gDNA) samples. In addition, internal control genes are also used to investigate abnormalities in gene number, and amplified oncogenes have been shown to have diagnostic, prognostic, and therapeutic relevance. Thus, TaqMan PCR-based gene quantification assays are also used to identify allelic imbalances (germ-line deletions or amplifications), for example, in individuals suffering from breast cancer, cutaneous melanoma, or nervous system tumors [8, 9].

Glyceraldehyde-3-phosphate dehydrogenase (GAPDH) represents a universally expressed reference gene that has many biological roles in addition to its function in glycolysis. A GAPDH assay has been developed for the accurate normalization of feline messenger RNA (mRNA) expression [10], and this assay was recently validated and compared to other potential feline mRNA reference gene assays [11]. Subsequently, the GAPDH assay was also applied as quality control to test for the integrity of the gDNA and the absence of PCR inhibitors [10, 12, 13]. One report suggested that a single copy of the GAPDH pseudogene is present in the feline genome and that the feline GAPDH assay can therefore be used to quantify cell number in feline samples [14]. However, no information on the exact position or the sequence of this GAPDH pseudogene was provided [14]. In contrast, a variable number of GAPDH pseudogenes has been reported for other organisms [15, 16].

Pseudogenes for many different genes have been found in all animal genomes studied so far [17]. Due to the high sequence similarity of the pseudogenes to their “parent” gene, pseudogenes often interfere with PCR or hybridization experiments that are intended to detect the genes only [18, 19]. Specifically, processed pseudogenes typically lack introns and are therefore well known to hamper data interpretation in mRNA transcription analysis [18, 20]. Promising clinical applications of RT-PCR assays for the purpose of early diagnosis and relapse monitoring of micrometastatic tumor cells have suffered from false-positive results due to their interference with corresponding pseudogenes in the past [19]. Thus, the aim of this study was to (i) investigate the number and sequence(s) of the GAPDH pseudogene(s) in the feline genome, with a specific focus on the GAPDH assay region that is frequently used to normalize genome equivalents [14] and (ii) provide a suitable alternative qPCR system for the normalization of the amount of input gDNA and the determination of cell number in feline samples.

## 2. Materials and Methods

### 2.1. Sample Description

All cats included in this study were in experimental studies officially approved by the Veterinary Office of the Swiss Canton of Zurich (TVB 30/2003, 59/2005, and 99/2007). The cats were kept in groups under optimal ethological conditions in a barrier facility, as previously described [21]. All cats were euthanized for reasons unrelated to the present study.

Tissue samples were collected upon necropsy from two feline leukemia virus-infected cats (cat B8, female 3.8 years old, and cat 15, neutered male, 1.3 years old). The cats underwent histopathological examination, and samples from the rectum, colon, spleen, and jejunum were collected. Tissues for histology were processed as described [22], histologically examined, and verified to be free of pathological abnormalities. The samples for molecular analysis were snap frozen in liquid nitrogen following collection and stored at −80°C until nucleic acid extraction. 

### 2.2. Nucleic Acid Extractions

Tissues (approximately 25 mg) were homogenized prior to extraction in 180 *μ*L ATL buffer (Qiagen, Hombrechtikon, Switzerland) for genomic DNA isolation. The samples were processed using the DNeasy Blood & Tissue Kit (Qiagen) following the manufacturer's recommendations. For all nucleic acid extractions, negative controls consisting of 100 *μ*L of phosphate-buffered saline were prepared with each batch to monitor cross-contamination.

### 2.3. GAPDH Pseudogene Sequencing

For the analysis of the GAPDH-like sequences of the domestic cat, gDNA was obtained from tissue samples from two cats (cat B8, rectum and colon, and cat 15, spleen and jejunum). 

Primers were designed using Primer Express software (Version 3, Applied Biosystems, Rotkreuz, Switzerland) based on the GAPDH mRNA sequence [23] to encompass the previously published TaqMan sequence [10]. PCR amplification of the GAPDH pseudogenes using the forward primer binding the exon 2 (GCGCCTGGTCACCAGGGCTGC; pb position: 39–59) and the reverse primer located on the exon 4 (GACTCCACAACATACTCAGCACCAGCATCAC; bp position: 287–257) resulted in an approximately 249 bp fragment. To ensure a high fidelity of amplification, the PCR was performed using Phusion polymerase and HF buffer (Finnzymes, Ipswich, UK), 500 nM of each primer, and 2 *μ*L of extracted gDNA in a final volume of 20 *μ*L. The cycling conditions were as follows: initial denaturation for 2 min at 98°C, followed by 40 cycles of 20 sec at 98°C, 30 sec at 70°C, and 20 sec at 72°C prior to a final elongation at 72°C for 10 min. The PCR products were separated by 2.5% agarose gel electrophoresis, excised from the gel, purified using the GenElute Gel Extraction Kit (Sigma-Aldrich, Buchs, Switzerland), and cloned into the pCRII-TOPO TA cloning vector (Invitrogen, Basel, Switzerland) according to the manufacturer's instructions. A total of 15 of the obtained clones were sequenced by Microsynth (Balgach, Switzerland).

The sequences were analyzed using Clone Manager Professional software version 7.01 (Scientific & Educational Software, Cary, NC, USA).

### 2.4. Alternative qPCR Assay Design

In other species, albumin (ALB) is known to be a single-copy gene and therefore it has been used as an internal control gene [8, 9, 24, 25]. In order to determine whether feline ALB (fALB) is also present as a single-copy gene, sequence and gene organization information of the feline genome were retrieved from GenBank (no. NM_001009961) and Ensembl (ENSFCAG00000011854). Subsequently, a fALB qPCR assay was designed. The TaqMan hydrolysis probe and primers for the fALB assay were designed using Primer Express software (Version 2, Applied Biosystems; Table 1). The primer pair (Microsynth, Table 1) was tested to ensure that it amplified a product of the appropriate length using 5 *μ*L gDNA in a total volume of 25 *μ*L per reaction with an ABI Prism 7700 sequence detection system (Applied Biosystems) and the TaqMan Fast Universal PCR Master Mix (Applied Biosystems). The PCR products were analyzed by gel electrophoresis in a 3% agarose gel stained with ethidium bromide, and the bands were visualized using the ChemiGenius 2 Bio Imaging System (Syngene, Cambridge, UK).

The qPCR reactions were performed as described [11] from gDNA extracted from tissues (diluted 1 : 10). The thermocycling conditions consisted of initial denaturation at 95°C for 20 sec followed by 45 cycles of 95°C for 3 sec and 60°C for 45 sec. The gDNA was amplified and quantified using a Rotor-Gene 6000 real-time rotary analyzer (Corbett, Mortlake, VIC, Australia) and an ABI Prism 7700 sequence detection system (Applied Biosystems).

### 2.5. Production of a Standard for Absolute fALB Quantification

Cat gDNA was used to generate an fALB standard template for absolute quantification. A 150 bp sequence, consisting of the fALB TaqMan sequence, was amplified from 2 *μ*L target gDNA using the fALB primers (Table 1). The PCR reaction contained 2.5 units Taq DNA polymerase (Sigma), final concentrations of 250 nM of each primer, 200 *μ*M dNTPs (Sigma), 2 *μ*L 10 × PCR buffer (Sigma), and 2 *μ*L of template in a final volume of 20 *μ*L. The amplification was performed using a Biometra TPersonal thermal cycler (Biolabo, Châtel-St-Denis, Switzerland). The cycling conditions consisted of an initial denaturation at 94°C for 2 min followed by 40 cycles of 94°C for 30 sec, 55°C for 30 sec, and 72°C for 15 sec, with a final extension at 72°C for 2 min. The resulting amplicon was gel-purified and cloned into the TOPO TA cloning vector (Invitrogen). The inserts in selected clones were verified by sequencing using an ABI PRISM 310 genetic analyzer (Applied Biosystems), as described [26]. The fALB reference plasmid was linearized by restriction digestion using the enzyme *Sal*I (Roche, Rotkreuz, Switzerland) and gel-purified (Gen Elute PCR Clean-Up Kit, Sigma-Aldrich), and the copy number was determined spectrophotometrically (GeneQuant, Pharmacia Biotech) and by agarose gel electrophoresis (ChemiGenius 2 Bio Imaging System). Ten-fold serial dilutions of the standard templates in 30 *μ*g/mL carrier salmon sperm DNA (Invitrogen) were aliquoted and frozen at −20°C, as described [27].

### 2.6. Efficiency, Analytical Sensitivity, Linear Range, and Precision of the fALB qPCR Assay

The newly designed fALB qPCR assay was evaluated according to the MIQE guidelines [28] using the Rotor-Gene 6000 real-time rotary analyzer (Corbett). The efficiency of the assay was calculated as previously described [29], using the following equation: *E* = (10^(−1/slope)^) − 1. The analytical sensitivity of the system was defined by an endpoint dilution experiment using the ten-fold serially diluted standard template and ten replicates per dilution, as described [27]. The linear range of amplification of the fALB qPCR assay was determined by ten-fold serial dilution of the linearized standard template. For the precision analysis, a dilution of a standard template containing 10^5^ copies of fALB per reaction was chosen and assayed 10 and 13 times for the intrarun and interrun precision, respectively, and the mean value, standard deviation, and coefficients of variation were calculated. 

### 2.7. Nucleotide Sequence Accession Numbers

The sequence of the partial GAPDH pseudogene of the cat B8 sample (clone 14) was submitted to GenBank under accession number JX523658.

## 3. Results 

### 3.1. Investigation of the GAPDH Pseudogene Sequence

**Figure 1:**
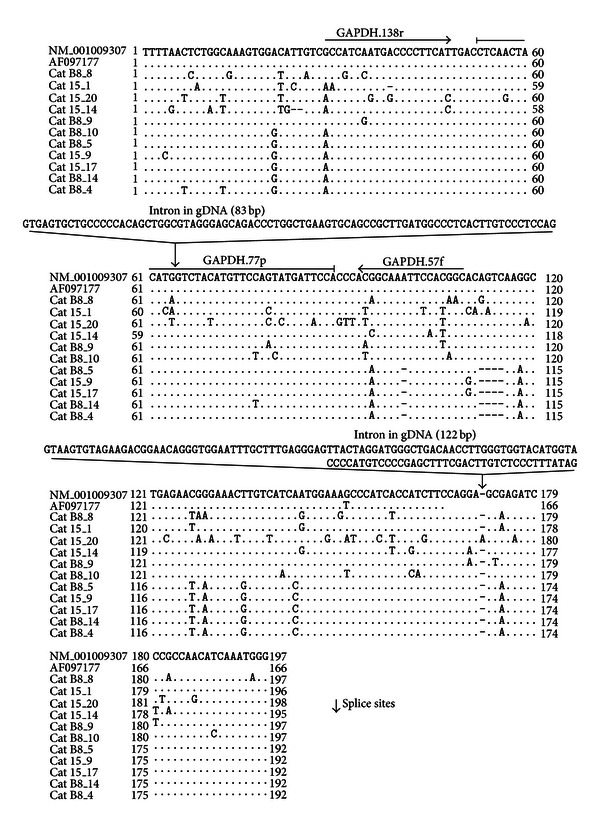
Comparison of feline GAPDH and pseudo-GAPDH nucleotide sequences. A comparison of the nucleotide sequences of feline GAPDH mRNA (GenBank: NM_001009307, position 60 to 256, and GenBank: AF097177, position 63 to 228) and gDNA sequences similar to GAPDH retrieved from cat B8 and cat 15. The number listed after the animal identifier (B8 and 15) represents the clone identity. Sequences found in multiple clones are shown only once. Nucleotides that differ from those in the two reference strains are indicated. Primer sequences for the qPCR reported previously [10] were located at positions 29 to 48 and 109 to 93, and the hydrolysis probe spans positions 53 to 89, as indicated. Arrows point to the splicing sites and the intron sequence from the gDNA (Ensembl: ENSFCAG00000006874) is depicted (Intron in gDNA). The exon sequences of the GAPDH gDNA (Ensembl: ENSFCAG00000006874) on which the GAPDH assay is located (exon 2 and 3) are 100% identical to the GAPDH mRNA sequence depicted in this figure (GenBank: NM_001009307).

An approach combining sequencing and bioinformatics was chosen to investigate the presence of potential GAPDH pseudogenes in the domestic cat genome. The potential target sequence of the GAPDH qPCR assay was amplified and cloned, and 15 clones were sequenced: 10 from cat B8 and 5 from cat 15. The sequencing of these 15 clones yielded 11 distinct sequences similar to that of the feline GAPDH mRNA (GenBank: NM_001009307). Eight different sequences were found within the target region of the primers and/or the hydrolysis probe of the GAPDH TaqMan assay (Figure 1). Overall, the sequence identity ranged from 82% (cat 15, clone 20: cat 15_20, Figure 1) to 96% (cat B8, clone 9: cat B8_9) in comparison to the reference sequence (GenBank: NM_001009307).

Sequence and gene organization information were retrieved from the Genome Annotation Resource Fields *Felis catus* v12.2 website (GARFIELD) http://lgd.abcc.ncifcrf.gov/ [30], and five sequences termed “similar to GAPDH” were found in the feline genome. One of these sequences on chromosome F2 exhibited 100% similarity to clone 14 of cat B8 (cat B8_14, Figure 1). In addition, the GAPDH assay sequence was searched using BLAST against the “AANG WGS Contigs” database containing the *Felis catus* whole-genome shotgun sequencing project (*Felis catus*-6.2; 14× coverage; [GenBank: AANG02000000]). Over 60 different contigs containing sequence similar to the GAPDH assay sequence were detected. The sequence with the best fit had three mismatches with the sequence of the GAPDH assay: two mismatches with the forward primer and one mismatch with the reverse primer.

### 3.2. Efficiency, Analytical Sensitivity, Linear Range, and Precision of the fALB qPCR Assay

In order to confirm the absence of pseudogenes for feline ALB, a bioinformatics approach was chosen. Sequence and gene organization information was retrieved from the GARFIELD website [30] and the second draft assembly, *Felis catus*-6.2 (GenBank: AANG02000000). Only one sequence termed “albumin” indicative of the albumin gene, was detected; it is located on chromosome B1.

Subsequently, a fALB real-time PCR assay was designed to amplify a sequence located within the exon IV of the feline albumin. The amplification of feline gDNA using the newly designed primers yielded PCR products with the expected size of 150 bp, as determined by agarose gel electrophoresis. The primers were then used in combination with the newly designed probe, and the amplification efficiency of the fALB qPCR was determined to be 99.5% to 100% using 10-fold serial dilutions of the standard template (Supplementary Material available online at http://dx.doi.org/10.1155/2013/587680 and data not shown). The highest dilution that still resulted in a positive signal in the qPCR assay contained an average of 1 copy of the standard in a 5 *μ*L reaction; in an endpoint dilution experiment, 6 of the 10 replicates of this dilution were positive. The fALB qPCR assay was linear over eight orders of magnitude, from 10^1^ to 10^8^ copies (Supplementary Material). The qPCR assay displayed a good precision; the coefficient of variation for the absolute number using 10^5^ copies/reaction was 5.51% for the intrarun precision analysis and 6.39% for the interrun analysis. 

## 4. Discussion

In this report, we describe the detection of several GAPDH-like sequences that are characteristic for processed GAPDH pseudogenes in the domestic cat genome. Based on the assumption that there is only one copy of the GAPDH pseudogene in the domestic cat genome [14], the GAPDH qPCR assay that was previously designed to amplify GAPDH mRNA/complementary DNA (cDNA) [10] was regularly used to determine the cell number of input gDNA. However, in our experience, the analysis of gDNA samples resulted in a lower amplification efficiency compared to cDNA (unpublished observations); we hypothesized that this might occur due to mismatches between the primers and/or hydrolysis probe and the gDNA sample. Thus, we performed a sequence analysis of the binding region of the primers and the hydrolysis probe of the GAPDH assay. The sequencing of 15 different clones comprising the GAPDH assay sequence revealed 11 different GAPDH-like sequences; however, none exhibited 100% similarity to the GAPDH mRNA sequence (GenBank: NM_001009307). It is possible that there are additional GAPDH pseudogenes present in the feline genome that were not detected in the present study because the sequencing was restricted to 15 clones. The use of an alternative sequencing technique (deep-sequencing rather than cloning followed by Sanger dideoxy sequencing) may provide a broader picture of the GAPDH-like sequences present in the cat genome. Furthermore, the primer binding sites chosen within the GAPDH sequence may have additionally restricted the number of recognized GAPDH pseudogenes. Thus, the number of GAPDH pseudogenes or GAPDH-like sequences in the feline genome may have been underestimated in our study. However, our sequencing results readily demonstrate that more than one feline GAPDH pseudogene is present in the genomic DNA of feline cells, and the GAPDH pseudogene sequences differed from the GAPDH mRNA sequence to some extent. 

This finding was supported by the sequence information retrieved from the GARFIELD website [30] and the second draft assembly, *Felis catus*-6.2 (GenBank: AANG02000000), in which no sequence with 100% similarity to the GAPDH mRNA sequence was found, but a multitude of closely related sequences were present. Moreover, our data are in agreement with studies investigating GAPDH pseudogenes in other species. In humans, between 56 and 62 GAPDH pseudogenes have been detected [15, 17]. Of note, the copy number of pseudogenes may vary within a population, as has been documented for the ATP-binding cassette transporter pseudogene within the Chinese population [31]. The number of recognized processed GAPDH pseudogenes may be as high as 120 in dogs and is over 300 in murine rodents [15–17]. For glycolytic genes, including GAPDH, it has been shown that there is a positive correlation between the level of gene expression and the abundance of processed pseudogenes in mice; GAPDH was found to be particularly highly expressed and to generate the highest number of pseudogenes among all glycolytic genes [17]. The authors explained the overabundance of GAPDH by the fact that GAPDH has many additional functions other than those related to glycolysis [17].

An alternative assay for the quantification of gDNA and cell number in feline samples was designed, validated, and implemented. In other species, different genes have been used for the quantification of cells, including the chemokine receptor CCR5 [32] and ALB [9]. ALB is known to be a single-copy gene in other species, such as humans and rhesus macaques, and has been used in both species as an internal control gene [8, 9, 24, 25]. According to the GARFIELD website [30], ALB is also present as a single-copy gene in the feline genome. ALB has been used for the normalization of retroviral provirus loads [24, 25, 33, 34]. We chose to develop a feline albumin assay because one of our group's main scientific interests is in retroviral infections. The newly implemented fALB assay was shown to be highly sensitive and efficient for gDNA, with a wide range of linearity. Our database searches resulted in only one hit termed “ALB.” Thus, to the best of our knowledge, fALB is a single-copy number gene.

## 5. Conclusions 

This study investigated the number of GAPDH pseudogenes in the domestic cat. Our results indicate that several closely related GAPDH-like sequences are indeed present in the cat genome. The GAPDH assay may still be used for quality control to test for the integrity of gDNA and the absence of PCR inhibitors. However, it appears to be a suboptimal choice for the quantification of gDNA equivalents. A newly designed assay using the fALB reference gene for the normalization of gDNA was validated and implemented. We recommend using this highly sensitive fALB qPCR assay for the normalization of input genomic DNA equivalents in future studies.

## Supplementary Material

Linearity of the qPCR assay and absolute quantification of fALB amplification: The linearity of the qPCR assay was determined using a ten-fold serial dilution of the linearized standard template. In this Figure, representative results from a qPCR run using a Rotor-Gene 6000 real-time rotary analyzer (Corbett) are shown. A) Amplification plot of the fALB qPCR assay depicts cycle number versus normalized fluorescence. B) A standard curve (of a representative qPCR) shows the logarithmic starting input quantity (copies per reaction) of a 10-fold serial dilution of the standard template versus the measured cycle threshold (CT). The CT refers to the number of cycles required before the fluorescence passes a fixed threshold. Earlier increases in normalized fluorescence are associated with lower threshold cycle numbers and therefore higher starting quantities of sample template. The fALB assay showed linearity over 8 orders of magnitude. The correlation coefficient of the curve was 0.999, and the slope of the dilution versus threshold cycle curve was -3.33, which is ideal.

## Figures and Tables

**Table 1:** Details of the TaqMan real-time PCR assays.

Gene	Oligo	Sequence (5′→ 3′)	Amplicon size (bp)	Final conc. (nM)
GAPDH^1^	Forward	GCCATCAATGACCCCTTCAT	82	480
	Reverse	GCCGTGGAATTTGCCGT		480
	Probe	CTCAACTACATGGTCTACATGTTCCAGTATGATTCCA^2^		160

ALB^3^	Forward	GATGGCTGATTGCTGTGAGA	150	500
	Reverse	CCCAGGAACCTCTGTTCATT		500
	Probe	ATCCCGGCTTCGGTCAGCTG^2,4^		200

^1^[10]; ^2^5′FAM/3′TAMRA; ^3^present study; ^4^HPLC purification.

## Abbreviations

qPCR: Quantitative real-time PCR
gDNA: Genomic DNA
GAPDH: Glyceraldehyde-3-phosphate dehydrogenase
fALB: Feline albumin
mRNA: Messenger RNA
cDNA: Complementary DNA
CT: Cycle threshold.

## References

[1] Kubista M, Andrade JM, Bengtsson M (2006). The real-time polymerase chain reaction. *Molecular Aspects of Medicine*.

[2] Bustin SA (2000). Absolute quantification of mRNA using real-time reverse transcription polymerase chain reaction assays. *Journal of Molecular Endocrinology*.

[3] Bernard PS, Wittwer CT (2002). Real-time PCR technology for cancer diagnostics. *Clinical Chemistry*.

[4] Mackay IM, Arden KE, Nitsche A (2002). Real-time PCR in virology. *Nucleic Acids Research*.

[5] Mackay IM (2004). Real-time PCR in the microbiology laboratory. *Clinical Microbiology and Infection*.

[6] Bustin SA, Mueller R (2005). Real-time reverse transcription PCR (qRT-PCR) and its potential use in clinical diagnosis. *Clinical Science*.

[7] Moreno LI, Tate CM, Knott EL (2012). Determination of an effective housekeeping gene for the quantification of mRNA for forensic applications. *Journal of Forensic Sciences*.

[8] Laurendeau I, Bahuau M, Vodovar N (1999). TaqMan PCR-based gene dosage assay for predictive testing in individuals from a cancer family with INK4 locus haploinsufficiency. *Clinical Chemistry*.

[9] Bieche I, Champème MH, Vidaud D, Lidereau R, Vidaud M (1998). Novel approach to quantitative polymerase chain reaction using real-time detection: application to the detection of gene amplification in breast cancer. *International Journal of Cancer*.

[10] Leutenegger CM, Mislin CN, Sigrist B, Ehrengruber MU, Hofmann-Lehmann R, Lutz H (1999). Quantitative real-time PCR for the measurement of feline cytokine mRNA. *Veterinary Immunology and Immunopathology*.

[11] Kessler Y, Helfer-Hungerbuehler AK, Cattori V (2009). Quantitative TaqMan® real-time PCR assays for gene expression normalisation in feline tissues. *BMC Molecular Biology*.

[12] Wolf-Jackel GA, Cattori V, Geret CP (2012). Quantification of the humoral immune response and hemoplasma blood and tissue loads in cats coinfected with “Candidatus Mycoplasma haemominutum” and feline leukemia virus. *Microbial Pathogenesis*.

[13] Novacco M, Boretti FS, Wolf-Jackel GA (2011). Chronic, “Candidatus Mycoplasma turicensis” Infection. *Veterinary Research*.

[14] Molia S, Chomel BB, Kasten RW (2004). Prevalence of Bartonella infection in wild African lions (Panthera leo) and cheetahs (Acinonyx jubatus). *Veterinary Microbiology*.

[15] Liu YJ, Zheng D, Balasubramanian S (2009). Comprehensive analysis of the pseudogenes of glycolytic enzymes in vertebrates: the anomalously high number of GAPDH pseudogenes highlights a recent burst of retrotrans-positional activity. *BMC Genomics*.

[16] Riad-El Sabrouty S, Blanchard JM, Marty L, Jeanteur P, Piechaczyk M (1989). The Muridae glyceraldehyde-3-phosphate dehydrogenase family. *Journal of Molecular Evolution*.

[17] McDonell L, Drouin G (2012). The abundance of processed pseudogenes derived from glycolytic genes is correlated with their expression level. *Genome*.

[18] Zhang Z, Carriero N, Gerstein M (2004). Comparative analysis of processed pseudogenes in the mouse and human genomes. *Trends in Genetics*.

[19] Ruud P, Fodstad O, Hovig E (1999). Identification of a novel cytokeratin 19 pseudogene that may interfere with reverse transcriptase-polymerase chain reaction assays used to detect micrometastatic tumor cells. *International Journal of Cancer*.

[20] Garbay B, Boue-Grabot E, Garret M (1996). Processed pseudogenes interfere with reverse transcriptase-polymerase chain reaction controls. *Analytical Biochemistry*.

[21] Geret CP, Riond B, Cattori V, Meli ML, Hofmann-Lehmann R, Lutz H (2011). Housing and care of laboratory cats: from requirements to practice. *Schweizer Archiv fur Tierheilkunde*.

[22] Helfer-Hungerbuehler AK, Cattori V, Boretti FS (2010). Dominance of highly divergent feline leukemia virus A progeny variants in a cat with recurrent viremia and fatal lymphoma. *Retrovirology*.

[23] Kullberg M, Nilsson MA, Arnason U, Harley EH, Janke A (2006). Housekeeping genes for phylogenetic analysis of eutherian relationships. *Molecular Biology and Evolution*.

[24] Dehée A, Césaire R, Désiré N (2002). Quantitation of HTLV-I proviral load by a TaqMan real-time PCR assay. *Journal of Virological Methods*.

[25] Chung HK, Unangst T, Treece J, Weiss D, Markham P (2008). Development of real-time PCR assays for quantitation of simian betaretrovirus serotype-1, -2, -3, and -5 viral DNA in Asian monkeys. *Journal of Virological Methods*.

[26] Willi B, Boretti FS, Cattori V (2005). Identification, molecular characterization, and experimental transmission of a new hemoplasma isolate from a cat with hemolytic anemia in Switzerland. *Journal of Clinical Microbiology*.

[27] Cattori V, Hofmann-Lehmann R (2008). Absolute quantitation of feline leukemia virus proviral DNA and viral RNA loads by TaqMan real-time PCR and RT-PCR. *Methods in Molecular Biology*.

[28] Bustin SA, Benes V, Garson JA (2009). The MIQE guidelines: minimum information for publication of quantitative real-time PCR experiments. *Clinical Chemistry*.

[29] Klein D, Janda P, Steinborn R, Müller M, Salmons B, Günzburg WH (1999). Proviral load determination of different feline immunodeficiency virus isolates using real-time polymerase chain reaction: influence of mismatches on quantification. *Electrophoresis*.

[30] Pontius JU, O’Brien SJ (2007). Genome annotation resource fields—GARFIELD: a genome browser for Felis catus. *Journal of Heredity*.

[31] Kringen MK, Stormo C, Grimholt RM, Berg JP, Piehler AP (2012). Copy number variations of the ATP-binding cassette transporter ABCC6 gene and its pseudogenes. *BMC Research Notes*.

[32] Thomas JA, Gagliardi TD, Alvord WG, Lubomirski M, Bosche WJ, Gorelick RJ (2006). Human immunodeficiency virus type 1 nucleocapsid zinc-finger mutations cause defects in reverse transcription and integration. *Virology*.

[33] Désiré N, Dehée A, Schneider V (2001). Quantification of human immunodeficiency virus type 1 proviral load by a TaqMan real-time PCR assay. *Journal of Clinical Microbiology*.

[34] Waters A, Oliveira ALA, Coughlan S (2011). Multiplex real-time PCR for the detection and quantitation of HTLV-1 and HTLV-2 proviral load: addressing the issue of indeterminate HTLV results. *Journal of Clinical Virology*.

