# Supplementary figures and images for: GAPDH Pseudogenes and the Quantification of Feline Genomic DNA Equivalents
Source: Mol Biol Int. 2013 Apr 28;2013:587680. doi: 10.1155/2013/587680 (PMC3655645; doi:10.1155/2013/587680)

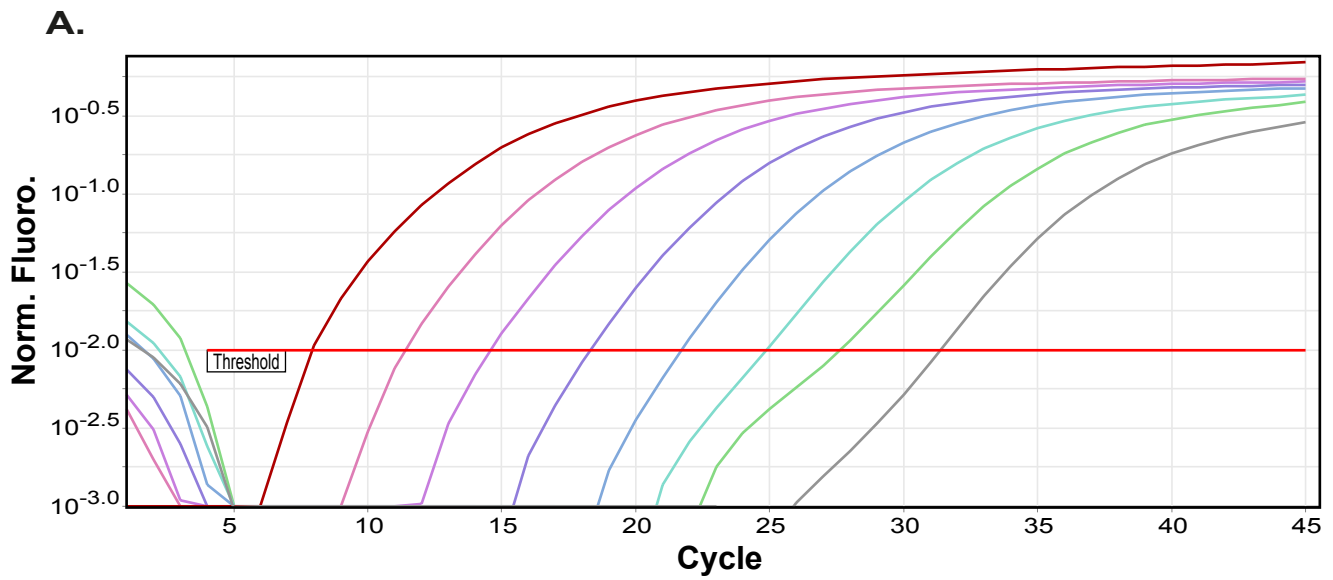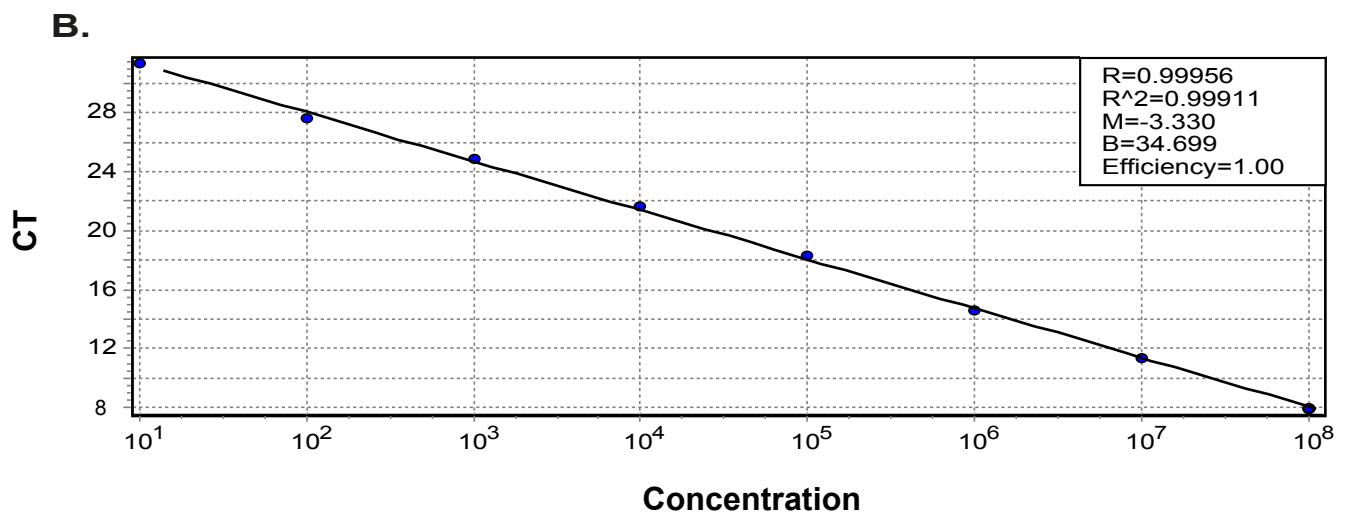

Supplement: Supplementary file 1 — Linearity of the qPCR assay and absolute quantification of fALB amplification: The linearity of the qPCR assay was determined using a ten-fold serial dilution of the linearized standard template. In this Figure, representative results from a qPCR run using a Rotor-Gene 6000 real-time rotary analyzer (Corbett) are shown. A) Amplification plot of the fALB qPCR assay depicts cycle number versus normalized fluorescence. B) A standard curve (of a representative qPCR) shows the logarithmic starting input quantity (copies per reaction) of a 10-fold serial dilution of the standard template versus the measured cycle threshold (CT). The CT refers to the number of cycles required before the fluorescence passes a fixed threshold. Earlier increases in normalized fluorescence are associated with lower threshold cycle numbers and therefore higher starting quantities of sample template. The fALB assay showed linearity over 8 orders of magnitude. The correlation coefficient of the curve was 0.999, and the slope of the dilution versus threshold cycle curve was -3.33, which is ideal. [file 587680.f1.pdf]
